# Sex differences in the genome-wide DNA methylation pattern and impact on gene expression, microRNA levels and insulin secretion in human pancreatic islets

**DOI:** 10.1186/s13059-014-0522-z

**Published:** 2014-12-03

**Authors:** Elin Hall, Petr Volkov, Tasnim Dayeh, Jonathan Lou S Esguerra, Sofia Salö, Lena Eliasson, Tina Rönn, Karl Bacos, Charlotte Ling

**Affiliations:** Epigenetics and Diabetes Unit, Department of Clinical Sciences, Lund University Diabetes Centre, CRC, Lund University, Scania University Hospital, 205 02 Malmö, Sweden; Islet Cell Exocytosis, Department of Clinical Sciences, Lund University Diabetes Centre, Lund University, CRC, 205 02 Malmö, Sweden

## Abstract

**Background:**

Epigenetic factors regulate tissue-specific expression and X-chromosome inactivation. Previous studies have identified epigenetic differences between sexes in some human tissues. However, it is unclear whether epigenetic modifications contribute to sex-specific differences in insulin secretion and metabolism. Here, we investigate the impact of sex on the genome-wide DNA methylation pattern in human pancreatic islets from 53 males and 34 females, and relate the methylome to changes in expression and insulin secretion.

**Results:**

Glucose-stimulated insulin secretion is higher in female versus male islets. Genome-wide DNA methylation data in human islets clusters based on sex. While the chromosome-wide DNA methylation level on the X-chromosome is higher in female versus male islets, the autosomes do not display a global methylation difference between sexes. Methylation of 8,140 individual X-chromosome sites and 470 autosomal sites shows sex-specific differences in human islets. These include sites in/near *AR*, *DUSP9*, *HNF4A*, *BCL11A* and *CDKN2B*. 61 X-chromosome genes and 18 autosomal genes display sex-specific differences in both DNA methylation and expression. These include *NKAP*, *SPESP1* and *APLN*, which exhibited lower expression in females. Functional analyses demonstrate that methylation of *NKAP* and *SPESP1* promoters *in vitro* suppresses their transcriptional activity. Silencing of *Nkap* or *Apln* in clonal beta-cells results in increased insulin secretion. Differential methylation between sexes is associated with altered levels of microRNAs *miR-660* and *miR-532* and related target genes.

**Conclusions:**

Chromosome-wide and gene-specific sex differences in DNA methylation associate with altered expression and insulin secretion in human islets. Our data demonstrate that epigenetics contribute to sex-specific metabolic phenotypes.

**Electronic supplementary material:**

The online version of this article (doi:10.1186/s13059-014-0522-z) contains supplementary material, which is available to authorized users.

## Background

Epigenetic factors such as DNA methylation are known to play important roles in tissue-specific gene expression, cell differentiation and parental imprinting. DNA methylation is also a key factor in X-chromosome inactivation, which takes place in all female mammalian cells to compensate for the extra X chromosome compared with male cells [1]. In mammalian cells, DNA methylation mainly takes place on cytosines in CG dinucleotides [2].

Sex differences at the DNA methylation level have previously been studied in some human tissues and cell types, such as blood, heart muscle and liver [3-7]. One study, analyzing LINE-1 and Alu repeats to investigate DNA methylation in blood, found a small but significantly higher degree of methylation in males compared with females [5]. Another study in human cell lines discovered that the active female X chromosome displayed similar DNA methylation patterns to that of the male X chromosome [8]. Moreover, CpG islands within promoter regions revealed higher methylation levels in the inactive compared with the active female X chromosome. In contrast, the body of multiple genes displayed lower methylation levels in the inactive compared with the active female X chromosome [8]. Analysis of sex differences in DNA methylation on the autosomal chromosomes have either revealed no, few or small changes [3,4,6,7]. However, in cells found in saliva, females tend to have higher DNA methylation levels on both the X chromosome as well as the autosomes [9]. Many of these studies have only analyzed a limited number of genes and gene regions, such as the promoter region, and have not performed genome-wide analyses of DNA methylation. In addition, sex-specific differences in DNA methylation levels have, to our knowledge, not yet been studied in human pancreatic islets.

DNA methylation is known to control the transcriptional activity differently depending on the genomic location of the methylation [10-12]. It is generally accepted that DNA methylation of gene promoters can be a source of gene silencing. Moreover, DNA methylation of the first exon was recently shown to be associated with decreased gene expression [10]. In contrast, a positive correlation between DNA methylation and gene expression has been demonstrated when methylation takes place in gene bodies [11,13-15], possibly because of stimulation of transcriptional elongation [11]. There are also data suggesting that tissue-specific and/or differential DNA methylation mainly occurs at CpG shores, and not in CpG islands [12,16].

Although previous studies have identified sex-specific differences in DNA methylation in, for example, saliva and blood [3,6,9], most of these studies have not linked epigenetic differences to differential gene expression and altered metabolism. However, sex differences in metabolism are well established and females have been shown to be more insulin sensitive and secrete more insulin compared with males [17-19], as measured by disposition index [17] or insulinogenic index [19], respectively. Nevertheless, this is a complex area and additional studies exploring the impact of sex on metabolic phenotypes are needed [20].

Based on these data, we hypothesize that sex-specific differences in DNA methylation may be associated with differential gene expression and altered insulin secretion in human pancreatic islets. The aim of this study was therefore to study the impact of sex on the genome-wide DNA methylation pattern in human pancreatic islets and relate this to sex-specific differences in gene expression, microRNA levels and insulin secretion.

## Results

### Impact of sex on glucose-stimulated insulin secretion in human pancreatic islets

The clinical characteristics of the 53 male and 34 female donors of human pancreatic islets are shown in Table 1. There were no significant differences in age, body mass index (BMI) or hemoglobin A1c (HbA1c) between the two sex groups. However, *in vitro* glucose-stimulated insulin secretion, measured as stimulation index (SI) [21], was higher in islets from females compared with males (Table 1). Additionally, we found no significant difference in islet purity (*P* = 0.27; data not shown) or β-cell content (*P* = 0.29; Additional file 1) between female and male islets.

**Table 1:** **Characteristics of all pancreatic islet donors divided by sex**

**Phenotype**	**Males**	**Females**	***P*** **-value**
n	53	34	
Age (years)	56 ± 11	58 ± 10	0.7
BMI (kg/m^2^)	25.6 ± 3.0	26.1 ± 4.1	0.7
Hba1c^a^ (%)	5.6 ± 0.5	5.6 ± 0.4	0.6
Stimulation index (SI^b^)	6.2 ± 5.8	7.6 ± 5.3	0.03

Data is expressed as mean ± standard deviation. *P*-values are based on Mann-Whitney test. ^a^Data available for 45 males and 28 females. ^b^Data available for 48 males and 33 females. BMI, body mass index; HbA1c, hemoglobin A1c.

### Sex differences in the genome-wide DNA methylation pattern in human pancreatic islets

**Figure 1:**
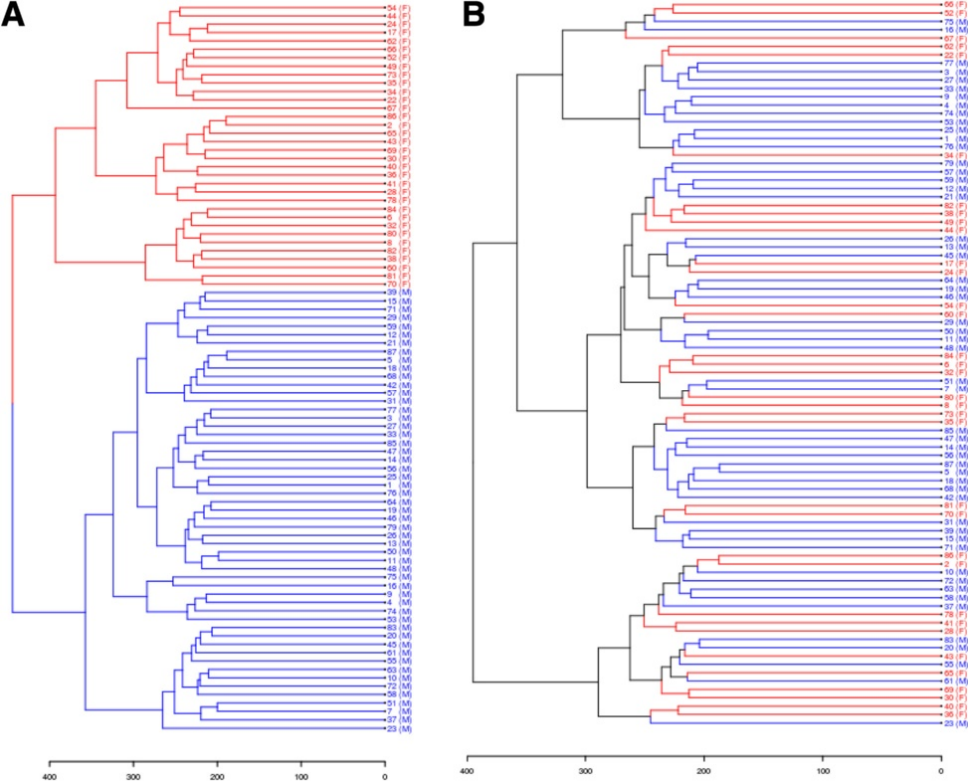
**Females and males cluster separately based on DNA methylation in human pancreatic islets. (A)** An unsupervised clustering analysis of the genome-wide DNA methylation data for all analyzed sites that passed quality control in human islets of 53 male and 34 female donors, after batch correction. The clustering includes 482,954 sites. Females are indicated with red color and (F), and males are indicated with blue color and (M). **(B)** An unsupervised clustering analysis of the genome-wide DNA methylation data including only autosomal data that passed quality control in human islets from 53 male and 34 female donors, after batch correction. Removal of X-chromosome DNA methylation data makes females and males cluster together. The clustering includes 471,804 sites. Females are indicated with red color and (F), and males are indicated with blue color and (M).

We next evaluated if there are epigenetic differences between sexes in human pancreatic islets. The genome-wide DNA methylation pattern was analyzed with the Infinium HumanMethylation450 BeadChip array. After quality control, DNA methylation data were obtained for a total of 482,954 sites, excluding data on the Y chromosome. We next performed an unsupervised clustering of the islet DNA methylation data for all 87 donors. Figure 1A clearly shows that the genome-wide DNA methylation data in human islets cluster based on sex. However, after removing the X chromosome methylation data (11,150 sites) from the data set we could no longer detect any clustering based on sex (Figure 1B).

**Figure 2:**
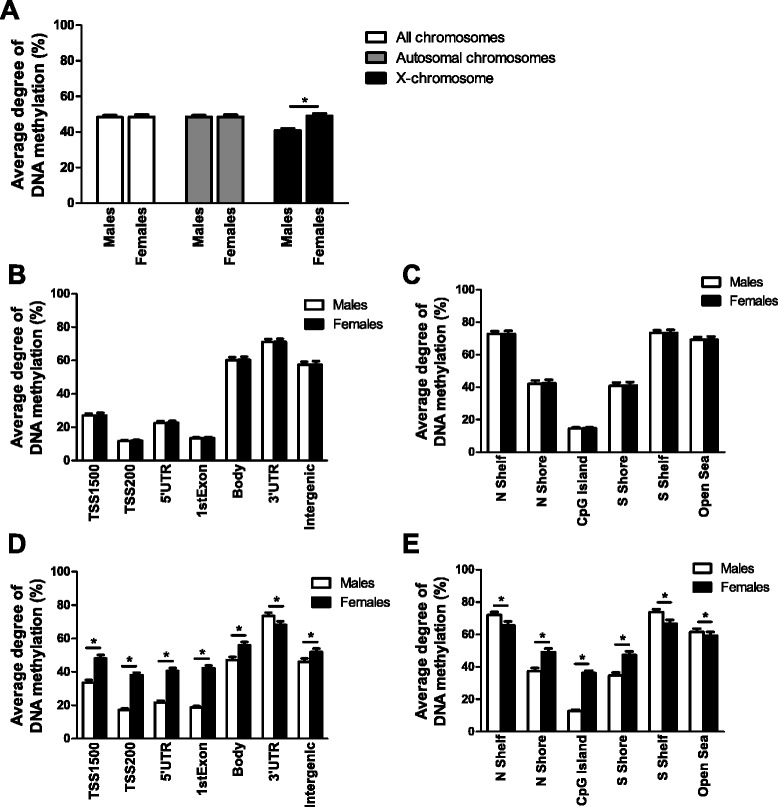
**Average degree of DNA methylation in human pancreatic islets from females and males. (A)** Average degree of DNA methylation of the sites analyzed using the Infinium HumanMethylation450 BeadChip array, including DNA methylation on the autosomal chromosome and the X chromosome but excluding methylation data on the Y chromosome, of human pancreatic islets from male and female donors. Data are presented as mean ± standard deviation. *P = 4.7 × 10^-15^. **(B,C)** Average DNA methylation levels of autosomal chromosomes for different functional genomic annotation **(B)** and CpG island regions **(C)** for human pancreatic islets from male and female donors. **(D,E)** Average DNA methylation levels of the X chromosome in gene regions **(D)** and CpG island regions **(E)** for human pancreatic islets from male and female donors. The 2 kb sequences, directly up- and downstream of CpG islands are called the northern and southern shore (N shore, S shore), respectively. The 2 kb sequences directly adjacent to the shores are called the northern and southern shelves (N shelf, S shelf). DNA methylation sites outside the CpG island regions are annotated as ‘open sea’. Data are presented as mean ± standard deviation. Asterisks indicate *q* <0.05 based on false discovery rate analysis. TSS, transcription start site.

We then tested if the average degree of DNA methylation of all analyzed sites differed between males and females. There were no differences in the average degree of methylation between the sexes when analyzing the DNA methylation sites on all chromosomes (females 48.5 ± 1.2% versus males 48.3 ± 1.1%, *P* = 0.48) or when analyzing the DNA methylation sites on the autosomal chromosomes separately (females 48.5 ± 1.2% versus males 48.5 ± 1.1%, *P* = 1.0) (Figure 2A). However, when analyzing the average degree of DNA methylation of all analyzed sites on the X chromosome, females exhibited a higher degree of methylation compared with males (females 48.9 ± 1.5% versus males 41.0 ± 1.1%, *P* = 4.7 × 10^-15^) (Figure 2A).

To further dissect the impact of sex on DNA methylation in human pancreatic islets, we calculated the average DNA methylation levels of the analyzed sites based on either their functional genomic annotation (TSS1500 (the region 200 to 1,500 nucleotides upstream of the transcription start site (TSS)), TSS200 (the 200 nucleotides immediately upstream of the TSS), 5′ untranslated region (UTR), first exon, body, 3′ UTR or intergenic) or the CpG content and neighborhood context [22]. This was done for the autosomal chromosomes and X chromosome separately. The average DNA methylation level of the studied genome regions did not differ significantly between males and females for the autosomal chromosomes (Figure 2B,C). For the X chromosome, however, all studied genome regions differed significantly between the sexes with false discovery rate (FDR) less than 5% (*q* <0.05) (Figure 2D,E). While the TSS1500, TSS200, 5′ UTR, first exon, body and intergenic regions had higher average methylation levels in females compared with males, the 3′ UTR region had a higher methylation level in males compared with females (Figure 2D). In addition, we found that females had higher average methylation levels compared with males for the shore regions as well as the CpG islands, while males had higher average methylation levels than females in the open sea and shelve regions (Figure 2E).

### Impact of sex on DNA methylation of individual sites in human pancreatic islets

**Figure 3:**
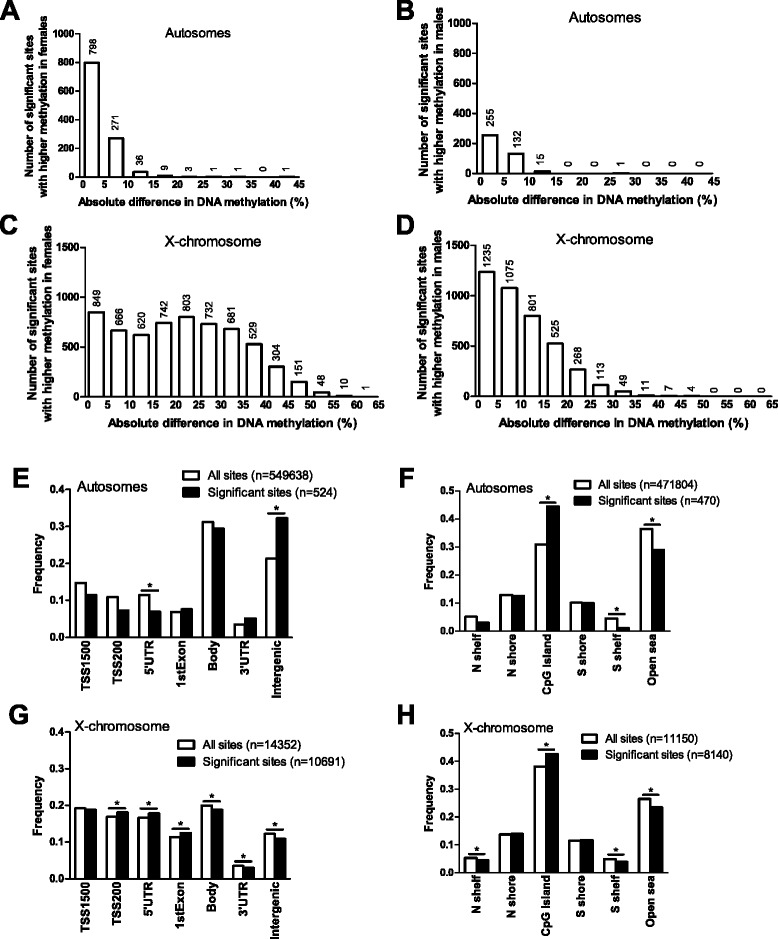
**Distribution of sites that exhibit differential DNA methylation between female and male pancreatic islets. (A-D)** The absolute difference in DNA methylation of individual sites with a significant difference in methylation between male and female pancreatic islets (*q* <0.05, based on an FDR analysis) divided into 5% bars. The absolute differences in DNA methylation of sites on the autosomal chromosomes with significantly higher methylation levels in females compared with males (n = 1,120) **(A)** or significantly higher methylation levels in males compared with females (n = 403) **(B)**. The absolute differences in DNA methylation of sites on the X chromosome with significantly higher methylation levels in females compared with males (n = 6,136) **(C)** or significantly higher methylation levels in males compared with females (n = 4,088) **(D)** based on FDR with *q* <0.05. **(E,F)** The distribution of significant sites (*q* <0.05, delta β-value >5%) versus all analyzed sites on the autosomal chromosomes in relation to genomic regions **(E)** and CpG island regions **(F)**. **(G,H)** The distribution of significant sites (*q* <0.05, delta β-value >5%) versus all analyzed sites on the X chromosome in relation to genomic regions **(G)** and CpG island regions **(H)**. Asterisks indicate significant (corrected *P*-value <0.05) based on a chi2 test of observed and expected significant and non-significant number of sites in each region. *P*-values in **(E-H)** have been corrected for multiple testing using Bonferroni correction.

Since it is known that individual CpG sites exhibit differences in DNA methylation between the sexes on both the autosomal chromosomes as well as the X chromosome in, for example, blood cells [3,5-7], we further tested if the degree of DNA methylation of the 482,954 analyzed sites in human pancreatic islets differed in males compared with females. The DNA methylation data on the autosomal chromosomes and the X chromosome were analyzed separately using a linear regression analysis including batch, age, BMI, purity of the islets, days in culture and HbA1c as covariates. We identified 1,523 individual sites on the autosomal chromosomes that exhibited significant differences in DNA methylation due to sex with FDR less than 5% (*q* <0.05). Figure 3A,B shows the number of significant sites (*q* <0.05) on the autosomal chromosomes distributed into 5% intervals of absolute difference in DNA methylation levels between males and females. To increase the biological relevance of our DNA methylation data, we further filtered the data to include only significant sites with absolute differences in DNA methylation bigger than 5% (delta β-value >5%) due to sex. We found 470 autosomal sites that had an absolute difference in DNA methylation bigger than 5% between the two sexes. These sites had a fold change (males/females) spanning between 0.22 and 3.86. Of these 470 sites, 322 had higher methylation in females and 148 sites had higher methylation in males. These 148 and 322 significant sites correspond to 82 and 140 individual genes, respectively (Additional files 2 and 3).

Furthermore, out of the total 11,150 sites analyzed on the X chromosome, 10,224 (92%) had a significant difference in DNA methylation based on sex with FDR less than 5% (*q* <0.05). These include 84 probes in non-CpG sites (that is, the C nucleotide is followed by an A or T). Figure 3C,D shows the distribution of the absolute difference in DNA methylation levels of the significant sites on the X chromosome between males and females. Out of these 10,224 sites, 8,140 had an absolute difference in DNA methylation of more than 5%. These 8,140 sites had a fold change (males/females) spanning between 0.041 and 6.43; 5,287 sites had a higher methylation in females compared with males and 2,853 had a higher methylation in males compared with females (Additional files 4 and 5). Out of these 8,140 sites, 31 were non-CpG sites, with 5 non-CpG sites being higher methylated in females compared with males and 26 non-CpG sites being higher methylated in males compared with females. The 5,287 sites on the X chromosome with >5% higher methylation in females correspond to 565 individual genes. The 2,853 sites on the X chromosome with >5% higher methylation in males compared with females correspond to 757 individual genes out of a total of 890 genes on the X chromosome covered by the Infinium HumanMethylation450 BeadChip. Of note, 450 of these X-chromosome genes have CpG sites with higher methylation in both females and males.

The distribution of autosomal and X-chromosome sites with a significant difference in DNA methylation >5% between males and females is shown in Figure 3E-H. The distribution is based on the site location in relation to the functional genomic annotation (Figure 3E,G) or in relation to CpG islands (Figure 3F,H). We found that CpG sites with a different level of DNA methylation on autosomal chromosomes were underrepresented within the 5′ UTR and enriched in intergenic regions (Figure 3E). Furthermore, the significant autosomal DNA methylation data also showed enrichment in the CpG island region, while they were underrepresented in the southern shelf and in the open sea region (Figure 3F). For the X chromosome, we found that the significant DNA methylation sites were enriched within TSS200, 5′ UTR and the first exon, while they were underrepresented in the gene body, 3′ UTR and in intergenic regions (Figure 3G). Also, the significant X-chromosome methylation data showed enrichment in the CpG island region and underrepresentation in the shelves and open sea region (Figure 3H).

Based on data by Chen *et al*. [23], we next tested if any of the Infinium probes that detected significant differences in DNA methylation between sexes in our cohort cross-react with alternative genomic locations. Three of the 113 possibly cross-reactive probes targeting the autosomal chromosomes (Additional file 6) have a perfect match to other locations in the genome, and 21 of the 346 possibly cross-reactive probes targeting the X chromosome have a perfect match to other locations in the genome (Additional file 7).

We also tested if any of the covariates included in the regression analysis had an impact on any of the individual sites that show significant differences in methylation >5% between sexes. Here, we found that only purity of the islets was significantly associated with DNA methylation of 78 autosomal sites and 642 sites on the X chromosome (Additional files 8 and 9).

### Biological features of the genes that exhibit differential methylation in human islets based on sex

We next performed a KEGG (Kyoto Encyclopedia of Genes and Genomes) pathway analysis using WebGestalt [24,25] to identify biological pathways with enrichment of genes that exhibit differential DNA methylation in human islets based on sex. The pathway analysis was conducted with genes differing significantly in DNA methylation by at least 5% due to sex on either the autosomal chromosomes (n = 220) or the X chromosome (n =872). The analysis of the autosomal chromosome genes was split into two separate analyses including either autosomal genes with a higher methylation in females or those higher in males. Two autosomal genes, *FLNB* and *TFDP1*, had a higher degree of DNA methylation, although at different CpG sites, in both females and males and these genes were subsequently included in the KEGG pathway analyses of genes with higher methylation in both sexes. Among the genes with higher methylation in females (n = 140) there was an enrichment of the cell adhesion molecule (CAM) pathway and among the genes with higher methylation in males (n = 82) there was an enrichment of the adipocytokine signaling pathway (Additional file 10). Pathway analysis on the X chromosome data did not generate any significantly enriched pathways.

**Figure 4:**
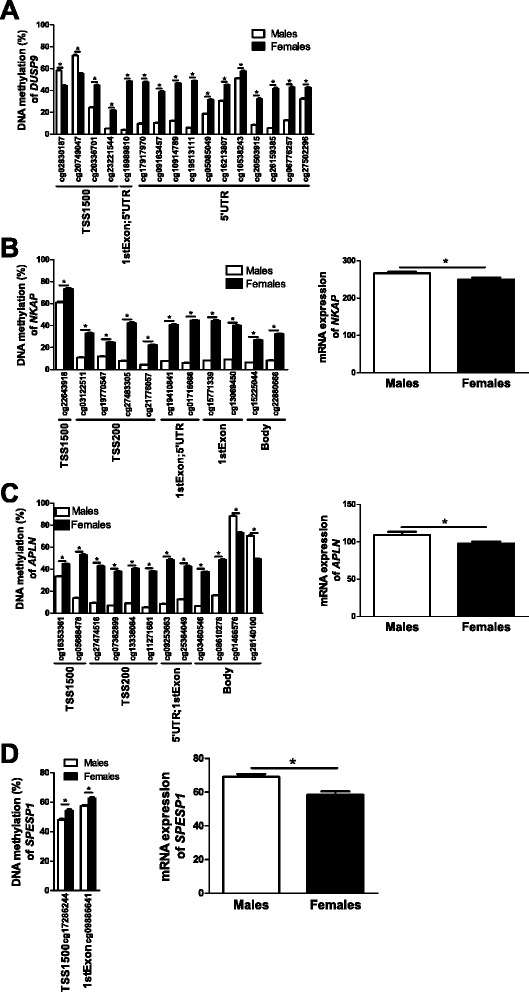
**Impact of sex on DNA methylation and/or expression in human pancreatic islets of selected candidate genes. (A)** *DUSP9*, a candidate gene for type 2 diabetes, is located on the X chromosome and has numerous differentially methylated CpG sites between female and male human pancreatic islets. **(B-D)** Impact of sex on the DNA methylation level of CpG sites and gene expression of *NKAP* **(B)**, *APLN* **(C)** and *SPESP1* **(D)**. Data are presented as mean ± standard error of the mean. Asterisks indicate *q* <0.05 for DNA methylation and *P* <0.05 for expression

Genome-wide association study (GWAS) data point to islet defects as key mechanisms in the development of type 2 diabetes and suggest that there are sex differences in the genetic predisposition to diabetes and related metabolic traits [26,27]. We therefore tested if there are sex differences in the degree of DNA methylation of candidate genes for type 2 diabetes and related traits in human pancreatic islets, which may contribute to altered metabolism and affect the risk for disease. We used gene lists of candidate genes for type 2 diabetes, and candidate genes for type 2 diabetes-related traits based on the online catalog of published GWAS [28]. Included genes had a *P*-value threshold for GWAS SNP finding at *P* <10^-5^. In total, the type 2 diabetes candidate gene list included 100 genes and 79 candidate genes for related traits. Sites annotated to 10 candidate genes for type 2 diabetes, including *DUSP9*, *BCL11A*, *HNF4A*, and *CDKN2B*, and 7 candidate genes for related metabolic traits (for example, *ATP11A*, *ADCY5* and *IRS1*) had differential DNA methylation in human pancreatic islets based on sex (Additional files 11 and 12). Interestingly, *DUSP9* is located on the X chromosome and had 16 CpG sites with an absolute difference in DNA methylation of more than 5% between males and females (Figure 4A).

Although most genes on one X chromosome are silenced in females, some genes are known to escape X-inactivation. We therefore checked if X-chromosome genes that show differential DNA methylation in male versus female pancreatic islets (Additional files 4 and 5) are previously known to escape X-chromosome inactivation based on experimental data in fibroblast-based systems from Carrel and Willard [29]. This analysis showed that out of the 565 X-chromosome genes containing sites with >5% higher methylation in islets from female compared with male donors, 209 were previously shown to be completely silent on the inactivated X chromosome, while 125 partially and 34 entirely escaped inactivation (Additional file 13). The remaining genes were not analyzed in the previous study [29]. For the 757 X-chromosome genes with sites that are more methylated in islets from male compared with female donors, 191 were previously shown to be completely inactivated, while 134 partially and 21 entirely escaped inactivation (Additional file 14).

### Sex differences in DNA methylation are associated with differential gene expression in human pancreatic islets

**Figure 5:**
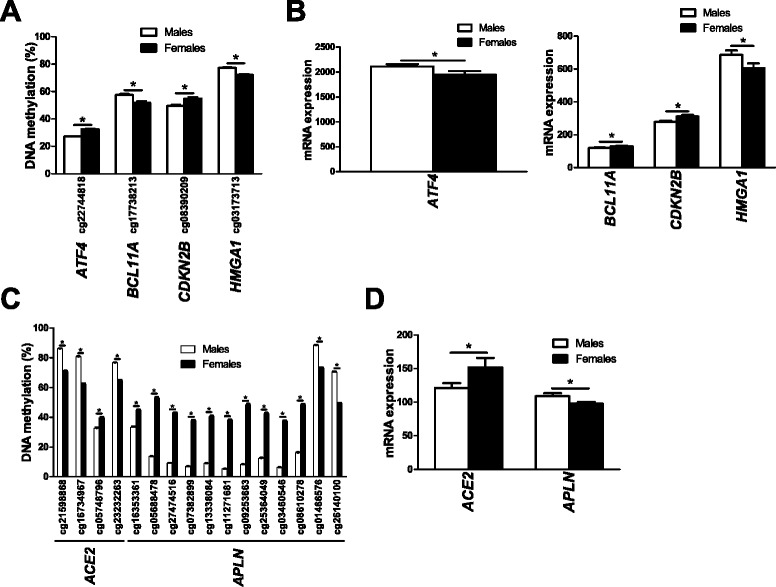
**Impact of sex on DNA methylation and expression in human pancreatic islets of genes previously associated with insulin secretion. (A-D)** DNA methylation and expression of genes with previously known function in insulin secretion located on autosomal chromosomes **(A**,**B)** or the X chromosome **(C**,**D)** in human pancreatic islets. Data are presented as mean ± standard error of the mean. Asterisks indicate *q* <0.05 for the DNA methylation data and *P* <0.05 for the gene expression data.

**Figure 6:**
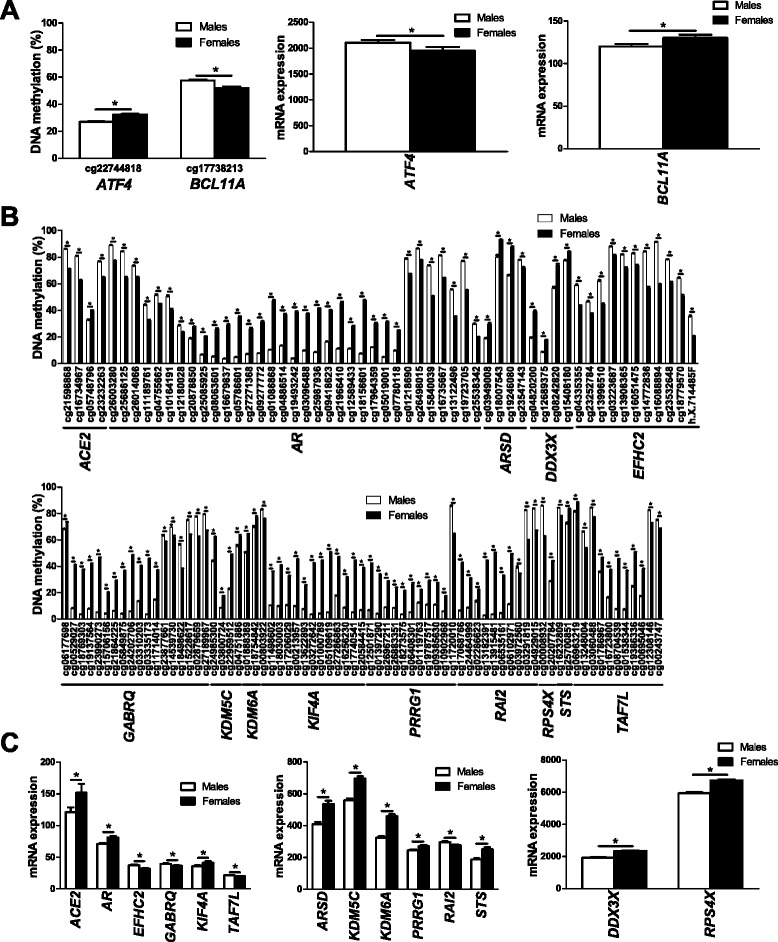
**Differential DNA methylation and expression of genes previously associated with sex differences. (A-C)** DNA methylation and gene expression of genes with previously shown sex differences located on autosomal chromosomes **(A)** or the X chromosome **(B,C)** in human pancreatic islets. Data are presented as mean ± standard error of the mean. Asterisks indicate *q* <0.05 for DNA methylation data and *P* <0.05 for gene expression data.

Since DNA methylation may regulate gene transcription [30,31], we further examined if any of the genes that exhibit differential DNA methylation due to sex also show different levels of expression in islets from male, compared with female, donors. Using microarray data, we identified 18 genes on the autosomal chromosomes (Additional files 15 and 16) and 61 genes on the X chromosome (Additional files 17 and 18) where differences in DNA methylation (*q* ≤0.05, absolute difference in DNA methylation >5%) were also associated with differential gene expression in human islets (*P* ≤0.05). These include genes previously known to promote NF-κB activation (that is, *NKAP*; Figure 4B) [32], to affect pancreatic islet function (for example, *APLN*; Figure 4C) [33], and to play a role during fertilization (that is, *SPESP1*; Figure 4D) [34]. Finally, based on literature search, genes with known function in the insulin secretion process and/or pancreatic islets function [33,35-39] (Figure 5) or with sex differences in normal or diseased tissues [40-51] (Figure 6) were found to be both differentially methylated and expressed between males and females.

### Sex differences in DNA methylation are associated with altered expression of microRNAs and their related target genes in human pancreatic islets

**Figure 7:**
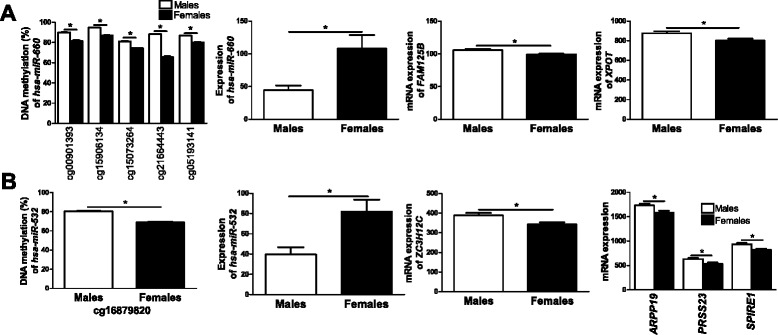
**Differential DNA methylation is associated with altered expression of microRNAs and their target genes in female compared with male human islets. (A)** Decreased DNA methylation of five CpG sites as well as increased expression (males n = 5, females n = 6) of *hsa-miR-660* in female compared with male islets*.* Also, potential target genes of *hsa-miR-660* showed lower gene expression in female compared with male human islets. **(B)** Decreased DNA methylation as well as increased expression (males n = 4, females n = 7) of *hsa-miR-532* in female compared with male islets. Also, potential target genes showed lower gene expression in female compared with male human islets. Data are presented as mean ± standard error of the mean. Asterisks indicate *q* <0.05 for DNA methylation data and *P* <0.05 for gene expression data.

DNA methylation of CpG sites annotated to microRNAs has been suggested to affect islet function in subjects with type 2 diabetes [52]. We therefore examined if CpG sites annotated to microRNAs exhibit differential DNA methylation in human pancreatic islets, based on sex. For autosomal chromosomes we found three microRNAs with significant differences in DNA methylation based on sex, namely *hsa-miR-548H4*, *hsa-miR-220B* and *hsa-miR-663B* (Additional files 2 and 3). Additionally, for the X chromosome, we found in total 160 sites annotated to microRNAs with differential DNA methylation between sexes, out of which 22 sites had higher methylation in females and 138 sites had higher methylation in males (Additional files 4 and 5). These sites correspond to six unique microRNAs with higher methylation in females and 59 unique microRNAs with higher methylation in males. Since DNA methylation may alter the expression of microRNAs and subsequently also the expression of their target genes [52], we further tested if any of the microRNAs that display differential DNA methylation due to sex also display altered expression in human islets. We found two microRNAs located on the X chromosome, *hsa-miR-660* and *hsa-miR-532*, that exhibited lower DNA methylation and higher expression levels in pancreatic islets from female compared with male donors (Figure 7). We next used TargetScan Human Release 6.2 (June 2012) [53] to find potential target genes of *hsa-miR-660* and *hsa-miR-532* (Additional files 19 and 20). Interestingly, six of the identified target genes showed lower expression in islets from female compared with male donors (Figure 7; Additional file 21).

### Technical validation of DNA methylation data in human pancreatic islets

**Figure 8:**
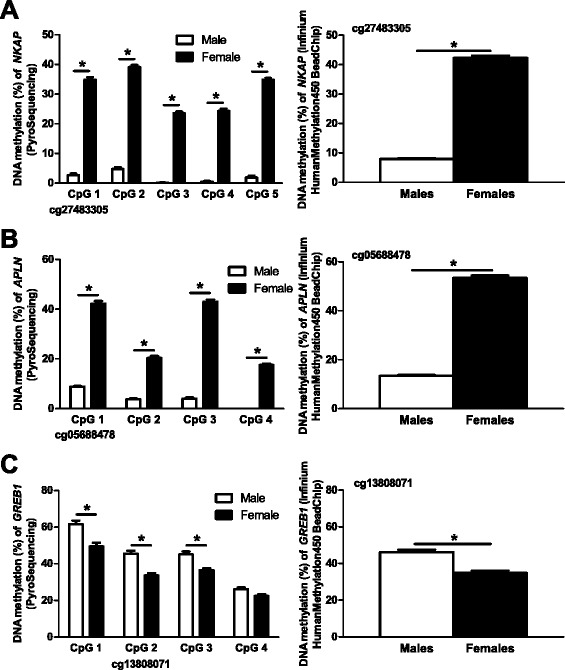
**Technical validation of Infinium HumanMethylation450 BeadChip data using PyroSequencing. (A-C)** The selected CpG site connected to *NKAP* (probe ID: cg27483305) **(A)**, *APLN* (probe ID: cg05688478) **(B)** and *GREB1* (probe ID: cg13808071) **(C)** showed similar differences in DNA methylation between males and females for both PyroSequencing and Infinium HumanMethylation450 BeadChip data. Numerous surrounding CpG sites in *NKAP* **(A)**, *APLN* **(B)** and *GREB1* **(C)** analyzed by PyroSequencing also show differential DNA methylation between male and female islet donors. Data are presented as mean ± standard error of the mean. Asterisks indicate *P* <0.0005 for PyroSequencing data and *q* <0.05 for Infinium HumanMethylation450 BeadChip data. PyroSequencing data were calculated using a Mann-Whitney test.

To technically validate the DNA methylation data from the Infinium HumanMethylation450 BeadChip, CpG sites annotated to *NKAP* (Figure 8A), *APLN* (Figure 8B) and *GREB1* (Figure 8C) were analyzed with PyroSequencing. These sites were chosen as they had relatively large differences in DNA methylation between males and females. DNA from pancreatic islets from 32 males and 18 females was used for the technical validation. In agreement with the Infinium HumanMethylation450 BeadChip data, all three CpG sites showed differential DNA methylation in pancreatic islets from male compared with female donors and the differences in DNA methylation seen with PyroSequencing were similar to the differences seen with the Infinium HumanMethylation450 BeadChip (Figure 8). Interestingly, we also found significant differences in DNA methylation between sexes for all but one adjacent CpG site only covered by the PyroSequencing assays, that is, sites not included on the Infinium HumanMethylation450 BeadChip (Figure 8A-C).

### Increased DNA methylation of the proximal promoters of *NKAP* and *SPESP1* decreases reporter gene expression

**Figure 9:**
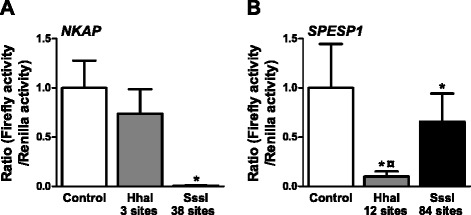
***In vitro*** **methylation of the** ***NKAP*** **and** ***SPESP1*** **promoters decreased reporter gene activity. (A,B)** The 1,500 bp promoter regions of *NKAP* **(A)** and *SPESP1* **(B)** were inserted into the pCpGL basic vector. Promoter constructs were then methylated (gray and black bars) with HhaI or SssI or mock-methylated (white bar) before transfection into clonal β-cells for 48 h. After the 48 h transfection, the luciferase assay was run. The data were normalized with co-transfected renilla luciferase control vector and are the mean of five (*NKAP*) or nine (*SPESP1*) separate experiments of five replicates in each. Cells transfected with an empty pCpGL vector were used as background control for firefly luciferase results, and untransfected cells were used as a background for renilla luciferase results. Data were log-transformed and statistical tests calculated using ANOVA followed by paired *t*-tests with Bonferroni correction *post hoc*. Data are presented as mean ± standard error of the mean. *P <0.05 versus control; ^¤^ *P* <0.05 versus SssI.

DNA methylation in the proximal promoter region is generally associated with decreased transcriptional activity [30,31]. We therefore functionally tested if promoter methylation affects expression of two selected genes that exhibited both differential DNA methylation and expression in islets due to sex (Figure 4B,D). While *NKAP* is located on the X chromosome, *SPESP1* is an autosomal gene located on chromosome 15. The promoter region of *NKAP* contains several differentially methylated CpG sites (*q* <0.05) and the mRNA expression of *NKAP* was lower in females compared with males (*P* <0.05) (Figure 4B). *SPESP1* has a higher degree of DNA methylation in the promoter and the first exon, in parallel with lower expression in female compared with male islets (Figure 4D). To functionally test if DNA methylation of the *NKAP* and *SPESP1* promoters could influence their gene expression, we used a luciferase reporter assay. A 1,500 bp DNA sequence upstream of their respective TSSs was inserted into a CpG free vector containing the firefly luciferase gene. The constructs were then either mock-methylated or methylated using two different enzymes, HhaI and SssI, where HhaI methylates the internal CpG site in the GCGC sequence, and SssI methylates all CpG sites in the sequence. The numbers of CpG sites methylated by these enzymes in the 1,500 bp *NKAP* and *SPESP1* promoter sequences are shown in Figure 9. SssI methylation of the *NKAP* promoter almost completely repressed the transcriptional activity of the reporter gene, while HhaI methylation did not significantly affect the transcriptional activity (*P* = 0.065), probably due to the low number of GCGC sequences in the promoter sequence of *NKAP* (Figure 9A). For the *SPESP1* promoter, methylation with either enzyme caused a significant reduction in transcriptional activity, with methylation by HhaI having a stronger repressive effect on transcription than SssI (Figure 9B). It may seem surprising that methylation of fewer CpG sites by HhaI decreased the transcriptional activity of the *SPESP1* promoter more than methylation by SssI. To further resolve why methylation by HhaI had a stronger repressive effect than SssI, we analyzed which transcription factors and repressive factors may bind to the *SPESP1* promoter and tested if their binding motif may overlap with CpG sites methylated by HhaI or SssI. Using TFSearch [54] we found numerous putative binding sites for transcription factors with previously known repressive function in the *SPESP1* promoter (Additional file 22). While the binding sites for many of these repressive factors overlap with or are in the immediate vicinity of CpG sites methylated by SssI, only three of these binding sites overlap with the GCGC sequence methylated by HhaI (Additional file 22). This may be an explanation for why methylation by HhaI had a stronger repressive effect on the transcriptional activity of *SPESP1* than SssI - that is, methylation of CpG sites where repressive factors bind may result in higher gene transcription.

### Silencing of *Nkap*, *Apln* and *Bcl11a* in clonal β-cells affects insulin secretion

**Figure 10:**
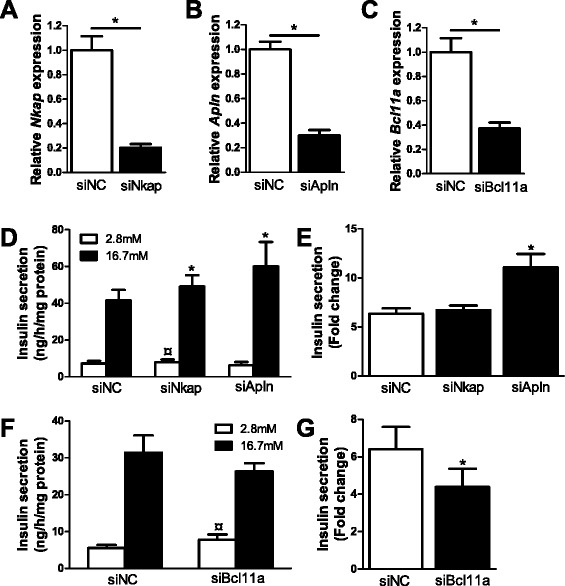
**Impact of** ***Nkap*** **,** ***Apln*** **and** ***Bcl11a*** **on insulin secretion in clonal β-cells.** Glucose-sensitive clonal INS-1 832/13 β-cells were used to study the impact of *Nkap*, *Apln* and *Bcl11a* on insulin secretion. **(A-C)** Transfection of clonal β-cells with siRNA targeting *Nkap*, *Apln* and *Bcl11a* resulted in decreased *Nkap* **(A)**, *Apln* **(B)** and *Bcl11a* **(C)** mRNA expression (black bars) when compared with clonal β-cells transfected with negative control siRNA (siNC, white bar) (n = 4). *P <0.05 using a Mann-Whitney test. **(D)** Insulin secretion in response to 2.8 mM (white bars) and 16.7 mM (black bars) glucose in clonal β-cells deficient for *Nkap* (n = 10) or *Apln* (n = 6) expression compared with control cells transfected with negative control siRNA (siNC) (n = 10). *P <0.05. Also, basal insulin secretion at 2.8 mM glucose showed a slight increase in si*Nkap*-treated cells compared with control (^¤^ *P* = 0.049). **(E)** Glucose-stimulated insulin secretion presented as the ratio of secretion at 16.7 over that at 2.8 mM glucose (fold change) in clonal β-cells deficient for *Nkap* (n =10) or *Apln* (n = 6) expression (black bars) compared with control cells transfected with negative control siRNA (siNC, white bar) (n = 10). **(F)** Insulin secretion in response to 2.8 mM (white bars) and 16.7 mM (black bars) glucose in clonal β-cells deficient for *Bcl11a* (n = 9) expression compared with control cells transfected with negative control siRNA (siNC) (n = 9). *P <0.05. **(G)** Glucose-stimulated insulin secretion presented as the ratio of secretion at 16.7 over that at 2.8 mM glucose (fold change) in clonal β-cells deficient for *Bcl11a* (n = 9) expression (black bars) compared with control cells transfected with negative control siRNA (siNC, white bar) (n = 9). *P <0.05. The insulin secretion data were normalized to total protein content. Insulin secretion data were analyzed using a Wilcoxon test. Data are presented as mean ± standard error of the mean.

The identified sex differences in islet DNA methylation and gene expression may contribute to the higher glucose-stimulated insulin secretion seen in pancreatic islets from females compared with males (Table 1; Additional files 15, 16, 17 and 18). To model the situation in human islets and elucidate whether altered expression of identified genes may affect insulin secretion, we used small interfering RNA (siRNA) to silence *NKAP* and *APLN*, two X-chromosome genes that showed increased promoter DNA methylation and decreased expression in female islets (Figure 4B,C and Figure 10), in clonal β-cells. We also silenced an autosomal gene, *BCL11A* (Figure 5A,B and Figure 10), that showed decreased DNA methylation and increased expression in female islets in the clonal β-cells. This resulted in approximately 80%, 70% and 60% reductions of *Nkap*, *Apln* and *Bcl11a* levels, respectively (Figure 10A-C). Glucose-stimulated insulin secretion at 16.7 mM glucose increased in clonal β-cells deficient for either *Nkap* or *Apln* expression. In addition, basal insulin secretion at 2.8 mM glucose increased in β-cells deficient for *Nkap* or *Bcl11a* expression (Figure 10D,F). Subsequently, the fold change of insulin secretion, calculated as the ratio of secretion at 16.7 over that at 2.8 mM glucose, increased in clonal β-cells deficient for *Apln* expression (Figure 10E), while it decreased in cells deficient for *Bcl11a* (Figure 10G).

## Discussion

This study is, to our knowledge, the first to examine the impact of sex on the genome-wide DNA methylation pattern in humans using the Infinium HumanMethylation450 BeadChip and to describe sex differences in the methylome and transcriptome in human pancreatic islets. We identified both chromosome-wide and site-specific sex differences in DNA methylation on the X chromosome of human pancreatic islets. However, the autosomal chromosomes showed differences in DNA methylation only on the level of individual CpG sites between sexes. Importantly, we also found higher insulin secretion in pancreatic islets from females compared with males, as well as sex differences in gene expression, including microRNAs. Additionally, we did not find any difference in β-cell number between females and males. This suggests that the difference in insulin secretion could be due to pancreatic islet function rather than a difference in actual β-cell number. It also suggests that the DNA methylation differences seen between males and females are not due to altered β-cell composition in the islets.

DNA methylation is known to contribute to X chromosome inactivation in female mammalian cells [1]. However, in our study males displayed higher average DNA methylation levels in the 3′ UTR gene region, as well as in the northern and southern shelves of CpG islands and in the open sea, while females displayed a higher average methylation in the TSS1500, TSS200, 5′ UTR, first exon, gene body and intergenic regions as well as in shore regions and the CpG islands. Also, a previous study by Liu *et al*. [9] identified several CpG sites on the X chromosome with higher DNA methylation levels in cells from saliva from males. In their study they only analyzed DNA methylation in promoter regions, and hence no data on other gene regions was presented. We also found higher DNA methylation of specific CpG sites on the X chromosome in islets from male compared with female donors. Importantly, some of these genes - for example, *ACE2*, which encodes the Angiotensin I converting enzyme 2 - showed lower gene expression in islets from males compared with females. Differential expression of these genes may contribute to metabolic differences between sexes. We could not detect global sex differences for the average degree of DNA methylation on the autosomal chromosomes. However, we found numerous specific CpG sites on autosomal chromosomes with differences in DNA methylation between the sexes; hence, it seems like the sex differences in DNA methylation on the autosomal chromosomes are gene and/or site-specific rather than global.

Moreover, in this study we identified in total 18 genes on the autosomal chromosomes and 61 genes on the X chromosome with both differences in DNA methylation and gene expression between females and males. These include six genes (for example, *APLN*, *ATF4* and *HMGA1*) previously known to affect insulin secretion [33,36,39] and 16 genes (for example, *ARSD*, *KDM5C* and *KIF4A*) previously known to contribute to sex differences [42,46,48]. To investigate if any of these differences contribute to the lower insulin secretion seen in male compared to female islets, we chose to follow up two X chromosome genes, *APLN* and *NKAP*, and one autosomal gene, *BCL11A*, in functional studies. Indeed, silencing of these genes in clonal β-cells resulted in altered glucose-stimulated insulin secretion.

*APLN* encodes apelin, a peptide hormone that is widely expressed in several tissues such as the heart and adipose tissue [33,55]. It is also known to be expressed in both human and rodent islets [33]. Previous studies have shown that apelin decreases glucose-stimulated insulin secretion at physiological levels and it was also shown to be expressed at higher levels in pancreatic β-cells of diabetic animal models compared with wild-type animals [33]. Additionally, it has been demonstrated that reduced insulin secretion in the presence of apelin is coupled to reduced levels of cAMP through effects on PDE3B [56]. The results from previous studies are in line with our data, since we found decreased DNA methylation in parallel with increased *APLN* expression in male islets, which secrete less insulin compared with female islets. Moreover, *APLN* was previously shown not to escape X-chromosome inactivation [29]. In the present study we also found that silencing of the apelin gene (*Apln*) in clonal β-cells increased glucose-stimulated insulin secretion.

*NKAP* encodes the NF-κB activating protein, which is a nuclear protein involved in the activation of the transcription of NF-κB [32]. NF-κB is known to positively regulate the transcription of several inflammatory cytokines, such as IL-1β, interferon-γ and TNF-α [57], which have been shown to decrease glucose-stimulated insulin secretion [58-60]. This could provide a possible mechanism for increased insulin secretion in *Nkap* silenced cells and in islets that exhibit higher methylation and lower expression of *NKAP*, for example, female islets. Furthermore, methylation of the *NKAP* promoter in clonal β-cells *in vitro* resulted in decreased transcriptional activity of the reporter gene. This could imply that there is a similar mechanism for transcriptional regulation of *NKAP in vivo* and that increased DNA methylation contributes to decreased *NKAP* expression in the human islets. Together, our data suggest that DNA methylation plays a role not only in regulating the differential expression between males and females, but could also be a possible explanation for the differential insulin secretion between sexes. Additionally, *NKAP* has previously been shown to escape X-chromosome inactivation in a fraction of cells [29].

*BCL11A* encodes B-cell CLL/lymphoma 11A, a zinc finger protein essential for lymphoid development [61,62] as it controls the expression and activity of *Bcl2*, *Bcl2-xL*, *Mdm2* and p53 and thereby cell survival. Furthermore, knockdown of *BCL11A* leads to increased rates of apoptosis in lymphoma cell lines [63-65]. Possibly, reduced *BCL11A* expression could also affect survival of other cell types, including β-cells, and thereby diabetes risk. Indeed, common variants in this gene have previously been associated with an increased risk of type 2 diabetes and with altered pancreatic islet hormone secretion [37,66-68]. Additionally, Tang and co-workers [69] recently showed that blood cells from males with type 2 diabetes had increased methylation of *BCL11A* compared with healthy male controls. In the present study, we found increased DNA methylation and decreased mRNA expression of *BCL11A* in male compared with female pancreatic islets. We also found a lower fold change of insulin secretion after silencing *Bcl11a* in clonal β-cells, indeed supporting a functional role of this gene in pancreatic β-cells.

Other identified genes that might help explain the difference in insulin secretion between females and males are *ACE2*, *ATF4* and *TFE3*. In our study, islet expression of *ACE2*, which encodes the Angiotensin I converting enzyme 2, was 25% higher in females than in males and this protein has previously been shown to have positive effects on β-cell proliferation and insulin secretion [70,71]. It was found that pancreatic overexpression of *Ace2* in hyperglycemic *db/db* mice resulted in increased insulin secretion while having no effect on insulin sensitivity [70]. Furthermore, islet insulin content and β-cell mass were also increased and together these changes resulted in a lowered blood glucose in the hyperglycemic *db*/*db* mice. The expression of *ATF4*, encoding cyclic AMP-dependent transcription factor ATF4, was higher in male islets and this protein is known to have negative effects on insulin secretion via both direct and indirect effects on the β-cell [72-74]. Through an interaction with FoxO1 in osteoblasts, ATF4 causes a decrease in circulating levels of the active form of the insulinotropic hormone osteocalcin, which in turn results in a reduction in pancreatic islet number, β-cell mass and insulin secretion and a worsened glucose tolerance [72,73]. In β-cells, ATF4 interacts with TRB3 and lowers the expression of key exocytosis genes [74]. Furthermore, the expression of *TFE3* was higher in male islets together with differential DNA methylation in several CpG sites connected to this gene. *TFE3* encodes transcription factor E3, which is known to stimulate expression of genes downstream of transforming growth factor-beta signaling. Since transforming growth factor-beta singling affects insulin secretion negatively, this could provide a possible mechanism for the lower insulin secretion in males compared with females [75].

Differences in DNA methylation on the X chromosome are expected between males and females. The difference in gene expression found in our study is more surprising. However, it is known that approximately 15% of all X-chromosome genes consistently escape X-chromosome inactivation and an additional 10% of these genes show a variable degree of X-chromosome inactivation escape, and may do so in a tissue-specific manner [29]. Subsequently, some of the genes identified in our study may escape X-chromosome inactivation. Indeed, data from Carrel and Willard [29] support the escape of X-chromosome inactivation of some of the genes identified to be differentially methylated in the present study. Additionally, this makes the genes that show differences in both DNA methylation and gene expression between the sexes of interest for further studies in order to fully understand differences between males and females.

Interestingly, we found several CpG sites annotated to microRNAs with differential DNA methylation between males and females. Two of these microRNAs, *hsa-mir-660* and *hsa-miR-532*, also showed elevated expression in parallel with decreased DNA methylation in female compared with male islets. Although no previous study has linked these microRNAs to islet function, we found sex differences in islet expression of six target genes for *hsa-miR-660* and *hsa-miR-532* (for example, *XPOT* and *SPIRE1*). Furthermore, studies have shown a sex-specific response to different pathological conditions and it has been discussed whether microRNAs could play a role in this sex-specific response [76]. However, to our knowledge, this is the first study showing sex differences in DNA methylation and expression of microRNAs together with target genes in human pancreatic islets.

When specifically analyzing if candidate genes for type 2 diabetes and related metabolic traits showed any differences in DNA methylation between sexes, we found that 10 of the type 2 diabetes candidate genes and 7 of the type 2 diabetes trait candidate genes had significant differences in DNA methylation between males and females. Several of these genes (for example, *HNF4A* and *CDKN2B*) have been proposed to be associated with pancreatic islet function [38,77] and it is hence possible that they contribute to sex differences in islets function.

It is important to be aware that the Infinium HumanMethylation450 BeadChip array includes probes with cross-reactivity to other sites in the genome [23,78]. This presents a particular difficulty when studying sex differences with autosomal probes that possibly could cross-react to the X or Y chromosome. However, in this study we only found a few cross-reactive probes with a perfect match to another genomic location.

## Conclusion

Our study has identified both chromosome-wide and gene-specific sex differences in DNA methylation on the X chromosome of human pancreatic islets, whereas the autosomal chromosomes only showed site-specific differences. These epigenetic differences were associated with differential gene expression, microRNA levels and insulin secretion in human pancreatic islets. Our functional studies further support that altered expression of these genes might explain secretory differences *in vivo*.

## Materials and methods

### Human pancreatic donors

Pancreatic islets from 87 deceased non-diabetic donors were obtained from the human tissue laboratory at Lund University Diabetes Centre (Table 1). Only islets from non-diabetic individuals with an HbA1c below 6.5% were included in this study. HbA1c was measured using the mono-s method [79]. Islets were prepared by collagenase digestion and density gradient purifications and they were cultured for 4 ± 2 days as previously described prior to DNA and RNA extraction [80]. The purity of the islet preparations was determined by dithizone staining.

Glucose-stimulated insulin secretion, calculated as SI, from the human islets was measured *in vitro* in static incubations as previously described [21]. Maximum and minimum SI values were set to 1 and 30, respectively, since this is more biologically relevant [21]. Informed consent for organ donation for medical research was obtained from pancreatic donors or their relatives in accordance with the approval by the regional ethics committee in Lund, Sweden (Dnr 173/2007). This study was performed in agreement with the Helsinki Declaration and in adherence to the regulations of the regional ethics committee.

### Calculating β-cell content

β-cell content in human islets of donors with available embedded islets (six male and seven female donors) was analyzed using transmission electron microscopy. Hand-picked islets were fixed in 2.5% glutaraldehyde in freshly prepared Millonig and post-fixed in 1% osmium tetroxide before being dehydrated and embedded in AGAR 100 (Oxford Instruments Nordiska, Lidingö, Sweden) and cut into ultrathin sections as previously described [81]. The sections were put on Cu-grids and contrasted using uranyl acetate and lead citrate. The islet-containing sections were examined in a JEM 1230 electron microscope (JEOL-USA. Inc., Peabody, MA, USA). Micrographs were analyzed for β-cell content using ImageJ and in-house software programmed in Matlab with methods previously described [82,83]. Islet cell types were distinguished by means of granular appearance: β-cell granules have a dense core surrounded by a white halo and α-cells have smaller dense granules lacking a distinguished halo. The ratio of β-cells in each islet was calculated by division of the total number of β-cells by the sum of the β-cell and α-cell numbers.

### DNA and RNA extraction

DNA and RNA were extracted from human pancreatic islets using the AllPrep DNA/RNA kit (Qiagen, Hilding, Germany) according to the manufacturer’s instructions. Nucleic acid purity and concentration were determined using a nanodrop (NanoDrop Technologies, Wilmington, DE, USA). All DNA samples had an A260/280 ratio of 1.8 to 2.1, whereas the 260/280 ratios for RNA were 1.9 to 2.2. The integrity and quality of the RNA were assessed using the Bioanalyzer (Agilent Technologies, Santa Clara, CA, USA) and available RNA integrity number (RIN) values from the Bioanalyzer were between 8.6 and 10.

### Genome-wide DNA methylation analysis of human pancreatic islets

Genome-wide DNA methylation analysis of human pancreatic islets was performed at the SCIBLU genomics centre at Lund University with the Infinium HumanMethylation450 BeadChip kit (Illumina, Inc., CA, USA). Genomic DNA (500 ng) was bisulfite converted using an EZ DNA methylation kit (Zymo Research, Orange, CA, USA). The total amount of bisulfite converted DNA was used to analyze DNA methylation with Infinium®assay using the standard Infinium HD Assay Methylation Protocol Guide (part number 15019519, Illumina). The bead chips were imaged using the Illumina iScan. The Infinium HumanMethylation450 BeadChip contains 485,577 probes out of which 3,091 are so-called non-CpG probes, and covers 99% of all RefSeq genes with the capacity for 12 samples per chip [22]. No probes on the chip are designed to target the pseudoautosomal region of the X chromosome, as these probes would not be unique. The GenomeStudio® methylation module software was used to calculate the raw methylation score for each DNA methylation site, which is presented as methylation β-value. The β-values are calculated as β = Intensity of the methylated allele (M)/(Intensity of the unmethylated allele (U) + Intensity of the methylated allele (M) +100). All samples passed GenomeStudio® quality control steps based on built-in control probes for staining, hybridization, extension and specificity and displayed high quality bisulfite conversion efficiency with an intensity signal above 4,000 [84]. Probes were then filtered based on Illumina detection *P*-value, and probes with a mean detection *P*-value >0.01 were removed from further analysis. In total, DNA methylation data were obtained for 483,031 probes out of which 3,039 probes are non-CpG sites. Since the cohort included islets from both males and females, Y-chromosome data were removed and subsequently DNA methylation data from 482,954 probes remained for further analysis. β-values were converted to M-values for further analysis (M = log2 (β/(1 - β)) [85] to remove heteroscedasticity in the data distribution. Background correction and quantile normalization were performed using the lumi package from bioconductor [86]. The methylation data were then separated on autosomal chromosomes and X chromosome before further analysis.

To identify differences in DNA methylation between males and females, the methylation data were analyzed using a linear regression model with the limma package in Bioconductor [87,88] including batch, age, BMI, purity of the islets, days in culture and HbA1c as covariates.

A FDR analysis was performed to correct *P*-values for multiple testing and *q*-values <0.05 were considered significant. In order to further test which factors affect the DNA methylation level in our dataset, we performed a principal component analysis of the DNA methylation data and correlated the top principal component with sex and all covariates of interest, including age, BMI, purity of the islets, days in culture and HbAa1c as described in [84]. Here, only sex and BMI showed significant correlations with the first principal component (*P* = 6.8 × 10^-3^ and *P* = 0.03, respectively). Since β-values are easier to interpret biologically, M-values were reconverted into β-values and were then used when describing the data and when generating figures. Non-CpG probes are presented with probe ID starting with ‘ch’ (Additional files 2, 3, 4, 5, 15, 16, 17, and 18). Moreover, since some probes on Illumina’s DNA methylation chip may cross-react to multiple locations in the genome, we used the published data by Chen *et al.* [23] to evaluate the number of possible cross-reactive probes among our significant methylation data (Additional files 6 and 7).

### mRNA expression analysis of human pancreatic islets

mRNA expression was analyzed using Affymetrix GeneChip® Human Gene 1.0 ST whole transcript based array (Affymetrix, Santa Clara, CA, USA) according to the manufacturer’s recommendations. We computed Robust Multichip Average (RMA) expression measure using the oligo package from Bioconductor [89]. To identify differences in gene expression between males and females, the data were analyzed using a linear regression model with the limma package in Bioconductor [87,88] with age, BMI, purity of the islets, days in culture, and HbA1c as covariates. We also performed a principal component analysis of the mRNA expression data and correlated the top principal component with sex and all covariates of interest, including age, BMI, purity of the islets, days in culture and HbAa1c as described in [84]. Here, purity of the islets showed significant correlations with the first principal component (*P* = 0.020), while sex and HbA1c showed significant correlations with the second principle component (*P* = 0.036 and *P* = 0.034).

### Locked nucleic acid-based microarray of human microRNAs

RNA from hand-picked human islets was extracted from four male and seven female donors using a miRNeasy kit (Qiagen). RNA quantity and quality were evaluated using spectrophotometry by Nanodrop and electropherogram profiles by Experion (BioRad, Hercules, CA, USA), respectively. Total RNA (500 ng) was directly labeled with miRCURY Hy3 fluorescent dye, which was subsequently hybridized to miRCURY LNA microRNA array v.11.0 in a Maui hybridization chamber according to the manufacturer’s recommendations (Exiqon, Vedbaek, Denmark). Images were acquired using Agilent array scanner and spot intensities were quantified in Genepix Pro 4.1. Array signals were normalized using the global Lowess regression algorithm as implemented in CARMAweb 1.4 [90]. For this study, only array signals from microRNAs within genomic loci showing differential methylation patterns were considered in the analysis.

### Validation of DNA methylation array results

Technical validation of the Infinium HumanMethylation450 BeadChip methylation data was performed by PyroSequencing (Qiagen) of three selected CpG sites (cg27483305, cg05688478, and cg13808071). Pre-designed PyroSequencing assays (PCR primers and sequencing primer) were used for the selected CpG sites (Qiagen) (Additional file 23). PyroSequencing was performed with the PyroMark™Q96 ID system (Qiagen) and all procedures were performed according to the manufacturer’s recommendations. In short, 500 ng genomic DNA from pancreatic islet of 50 human donors (32 males, 18 females) was bisulfite converted using EpiTect Bisulfite Kit (Qiagen), and 10 ng bisulfite converted DNA was then used as input for each PCR assay. Furthermore, PyroMark PCR Master Mix kit (Qiagen), streptavidin coated beads (GE Healthcare, Uppsala, Sweden), PyroMark Gold Q96 reagents (Qiagen) and PyroMark Q96 (version 2.5.8) software (Qiagen) were used for the analysis of DNA methylation, all according to the manufacturers’ recommendations.

### Luciferase assay

The luciferase assay protocol has been described in detail elsewhere [91]. Briefly, a 1,500 bp fragment of the *NKAP* or *SPESP1* promoter (Additional file 24) covering CpG sites that exhibited differential DNA methylation in female versus male islets (Figure 4) was cloned into a CpG-free luciferase reporter vector (pCpGL-basic) kindly provided by Dr Klug and Dr Rehli [92]. Amplification of the *NKAP* and *SPESP1* sequences and insertion into the pCpGL basic vector was performed by GenScript (GenScript USA Inc., Piscataway, NJ, USA). Two different DNA methyltransferases, SssI and HhaI (2.5 U/μg DNA; New England Biolabs, Frankfurt, Germany), were then used to methylate the constructs. SssI methylates all cytosine residues within the double-stranded dinucleotide recognition sequence CG and HhaI methylates only the internal cytosine residue in the GCGC sequence. The clonal rat β-cell line INS 832/13 was cultured as described [93], and co-transfected with 100 ng methylated or mock-methylated pCpGL vector including either the *NKAP* or *SPESP1* promoter insert together with 4 ng of pRL renilla luciferase control reporter vector (Promega, Madison, WI, USA). Firefly and renilla luciferase luminescence, as a value of transcriptional activity, was measured for each construct with the Dual-Glo® Luciferase Assay System (Promega) and an Infinite® M200 PRO multiplate reader (Tecan Group Ltd, Männedorf, Switzerland). The result represents the mean of at least five independent experiments, and the results of each experiment are the mean of five replicates. Cells transfected with an empty pCpGL vector were used as background control for the firefly luciferase results, and untransfected cells were used as a background for the renilla results.

### RNA interference of *Apln*, *Nkap* and *Bcl11a* in clonal β-cells

Three genes selected from the DNA methylation and mRNA expression analyses were silenced in clonal INS-1 832/13 β-cells [93] by siRNA transfection with Dharmafect I (Thermo Scientific, Waltham, MA, USA) according to the manufacturer’s instructions. The siRNAs (LifeTechnologies, Paisley, UK) used were s132851 (*Apln*), s151310 (*Nkap*), s156923 (*Bcl11a*) and a negative control siRNA (5′-GAGACCCUAUCCGUGAUUAUU-3′). RNA was isolated 72 h post-transfection with the RNeasy Plus mini kit (Qiagen) and subsequently converted to cDNA with the RevertAid First Strand cDNA Synthesis kit (Thermo Scientific). Knockdown was verified by quantitative PCR with the following TaqMan assays (Life Technologies); Rn00581093_m1 (*Apln*), Rn01297610_m1 (*Nkap*) and Rn01434083_m1 (*Bcl11a*). *Ppib* (Cyclophilin B; Rn03302274_m1) and *Hprt1* (Rn01527840_m1) were used as endogenous controls and quantification was done with the ΔΔCt method.

### Insulin secretion

Insulin secretion was determined during 1 h static incubations 72 h post-transfection of clonal INS-1 832/13 β-cells with siRNA against *Apln*, *Nkap* or *Bcl11a*. Prior to analysis of insulin secretion, clonal β-cells in multi-well plates were washed in HEPES balanced salt solution (HBSS; 114 mM NaCl, 4.7 mM KCl, 1.2 mM KH_2_PO_4_, 1.16 mM MgSO_4_, 20 mM HEPES, 2.5 mM CaCl_2_, 25.5 mM NaHCO_3_, 0.2% bovine serum albumin, pH 7.2) supplemented with 2.8 mM glucose and pre-incubated for 2 h at 37°C. Insulin secretion was then measured by static incubation for 1 h in 1.0 ml HBSS containing 2.8 or 16.7 mM glucose. Released insulin was determined with the Coat-a-Count Kit (Siemens Diagnostics, Erlangen, Germany) and normalized to the amount of protein in each well as measured with the BCA Protein Assay Kit (Thermo Scientific).

### Data availability

The Gene Expression Omnibus accession numbers for the methylation and expression array data reported in this paper are GSE62640 and GSE54279, respectively.

### Statistics

Unsupervised hierarchical clustering was performed with batch-corrected DNA methylation array data and calculated using the R software [94]. All data are presented as mean ± standard deviation unless stated otherwise. Phenotype data presented in the text or in Table 1 were calculated using a Mann-Whitney test in IBM SPSS statistics 20.0 (IBM Corp., released 2011, IBM SPSS Statistics for Windows, Version 20.0, Armonk, NY, USA).

## Additional files

Additional file 1:
**Sex does not affect the relative β-cell number in human pancreatic islets.** Analysis of β-cell number with transmission electron microscopy showed no difference between male and female human pancreatic islets. *P* =0.29 based on a Mann-Whitney U test. Data are presented as mean ± standard error of the mean. Additional file 2:
**Autosomal DNA methylation data (** ***q*** **<0.05, delta β-value >5%) with higher methylation levels in female compared with male islets.** Excel file showing significant DNA methylation sites with FDR less than 5% and absolute sex differences in DNA methylation bigger than 5%. Additional file 3:
**Autosomal DNA methylation data (** ***q*** **<0.05, delta β-value >5%) with higher methylation levels in male compared with female islets.** Excel file showing significant DNA methylation sites with FDR less than 5% and absolute sex differences in DNA methylation bigger than 5%. Additional file 4:
**X chromosome DNA methylation data (** ***q*** **<0.05, delta β-value >5%) with higher methylation levels in female compared with male islets.** Excel file showing significant DNA methylation sites with FDR less than 5% and absolute sex differences in DNA methylation bigger than 5%. Additional file 5:
**X chromosome DNA methylation data (** ***q*** **<0.05, delta β-value >5%) with higher methylation levels in male compared with female islets.** Excel file showing significant DNA methylation sites with FDR less than 5% and absolute sex differences in DNA methylation bigger than 5%. Additional file 6:
**Cross-reactive probes for autosomal chromosomes only.** Autosomal chromosome probes showing differential DNA methylation in human islets based on sex (*q* <0.05, delta β-value >5%) and with possible cross-reactivity to other locations in the genome. The map info is based on genome build 37. BLAT chromosome position, BLAT coordinates and numbers of matching base pairs are based on previously published data [23]. Of note, some probes cross-react to more than one location in the genome. Additional file 7:
**Cross-reactive probes for X chromosome only.** X chromosome probes showing differential DNA methylation in human islets based on sex (*q* <0.05, delta β-value >5%) and with possible cross-reactivity to other locations in the genome. The map info is based on genome build 37. BLAT coordinates and numbers of matching base pairs are based on previously published data [23]. Of note, some probes cross-react to more than one location in the genome. Additional file 8:
**Sites on the autosomal chromosomes where DNA methylation significantly associates with both pancreatic islet purity and sex.** Autosomal data where DNA methylation is significantly associated with both sex and the co-variate islet purity (*q* <0.05). Additional file 9:
**Sites on the X chromosome where DNA methylation significantly associates with both pancreatic islet purity and sex.** X-chromosome data where DNA methylation is significantly associated with both sex and the co-variate islet purity (*q* <0.05). Additional file 10:
**KEGG pathway analysis result.** Results from KEGG pathway analysis of genes with differential methylation in males and females for the autosomal chromosome genes. Additional file 11:
**Candidate genes for type 2 diabetes that exhibit differential DNA methylation in human pancreatic islets based on sex.** Excel file showing individual CpG sites located within/near candidate genes for type 2 diabetes [28], with a significant difference (*q* <0.05) in DNA methylation in human pancreatic islets between females and males. Additional file 12:
**Candidate genes for type 2 diabetes-related traits that exhibit differential DNA methylation in human pancreatic islets based on sex.** Excel file showing individual CpG sites located within/near candidate genes for type 2 diabetes-related traits [28], with a significant difference (*q* <0.05) in DNA methylation in human pancreatic islets between females and males. Additional file 13:
**X chromosome inactivation status of genes with sites that are more methylated in pancreatic islets from female compared with male donors.** Our significant methylation data were compared to data published by Carrel and Willard [29], where they studied the expression status of genes on the inactivated X chromosome in a fibroblast-based system. The list contains genes with sites that are more methylated in islets from female compared with male donors, separated into genes that do not or do, partially or completely, escape X chromosome inactivation based on the data by Carrel and Willard. Additional file 14:
**X chromosome inactivation status of genes with sites that are more methylated in pancreatic islets from male compared with female donors.** Our significant methylation data were compared to data published by Carrel and Willard [29] where they studied the expression status of genes on the inactivated X chromosome in a fibroblast-based system. The list contains genes with sites that are more methylated in islets from male compared with female donors, separated into genes that do not or do, partially or completely, escape X chromosome inactivation based on the data by Carrel and Willard*.*
Additional file 15:
**DNA methylation differences and expression differences in autosomal genes based on sex.** Excel file showing genes exhibiting both significantly higher DNA methylation in females compared to males (*q* <0.05, delta β-value >5%) as well as differential mRNA expression at *p* <0.05 in human pancreatic islets, for autosomal genes only. Additional file 16:
**DNA methylation differences and expression differences in autosomal genes based on sex.** Excel file showing genes exhibiting both significantly higher DNA methylation in males compared with females (*q* <0.05, delta β-value >5%) as well as differential mRNA expression at *P* <0.05 in human pancreatic islets, for autosomal genes only. Additional file 17:
**DNA methylation differences and expression differences in X-chromosome genes based on sex.** Excel file showing genes exhibiting both significantly higher DNA methylation in females compared with males (*q* <0.05, delta β-value >5%) as well as differential mRNA expression at *P* <0.05 in human pancreatic islets, for X-chromosome genes only. Additional file 18:
**DNA methylation differences and expression differences in X-chromosome genes based on sex.** Excel file showing genes exhibiting both significantly higher DNA methylation in males compared with females (*q* <0.05, delta β-value >5%) as well as differential mRNA expression at *P* <0.05 in human pancreatic islets, for X-chromosome genes only. Additional file 19:
**Target genes for** ***hsa-miR-660.*** Excel file showing potential target genes of *hsa-miR-660* generated using TargetScan Human release 6.2 (June 2012) [53]. Additional file 20:
**Target genes for** ***hsa-miR-532.*** Excel file showing potential target genes of *hsa-miR-532* generated using TargetScan Human release 6.2 (June 2012) [53]. Additional file 21:
**Differentially expressed target genes of** ***hsa-miR-660*** **and** ***hsa-miR-532.*** Excel file showing the differentially expressed target genes of *hsa-miR-660* and *hsa-miR-532* between female and male human islets. Additional file 22:
**Putative transcription factor binding sites in the** ***SPESP1*** **promoter.** List of transcription factors with known repressive effect and putative binding sites in the *SPESP1* promoter. Additional file 23:
**Assay information for technical validation of the Infinium HumanMethylation450 BeadChip (Illumina) DNA methylation data.** Table showing the assays used for technical validation of the Infinium HumanMethylation450 BeadChip (Illumina) using pre-designed PyroSequencing (Qiagen) assays. Position is based on genome build 37. Additional file 24:
**Sequences for the** ***NKAP*** **and** ***SPESP1*** **promoters used for luciferase assay.** We cloned 1,500 bp fragments of the *NKAP* and *SPESP1*promoters covering CpG sites that exhibited differential DNA methylation in female versus male islets into a CpG-free luciferase reporter vector for the luciferase assay.

## Abbreviations

BMI: body mass index
bp: base pair
FDR: false discovery rate
GWAS: genome-wide association study
HbA1c: hemoglobin A1c
HBSS: HEPES balanced salt solution
KEGG: Kyoto Encyclopedia of Genes and Genomes
SI: stimulation index
siRNA: small interfering RNA
SNP: single nucleotide polymorphism
TSS: transcription start site
UTR: untranslated region
