# Supplementary material for: Sex differences in the genome-wide DNA methylation pattern and impact on gene expression, microRNA levels and insulin secretion in human pancreatic islets
Source: Genome Biol. 2014 Dec 3;15(12):522. doi: 10.1186/s13059-014-0522-z (PMC4256841; doi:10.1186/s13059-014-0522-z)

Additional file 1: Sex does not affect the relative beta-cell number in human pancreatic islets. Analysis of beta cell number with transmission electron microscopy showed no difference between male (n=6) and female (n=7) islets (*p*=0.29). Statistics was calculated using Mann-Whitney. Data is presented as mean ± sem.

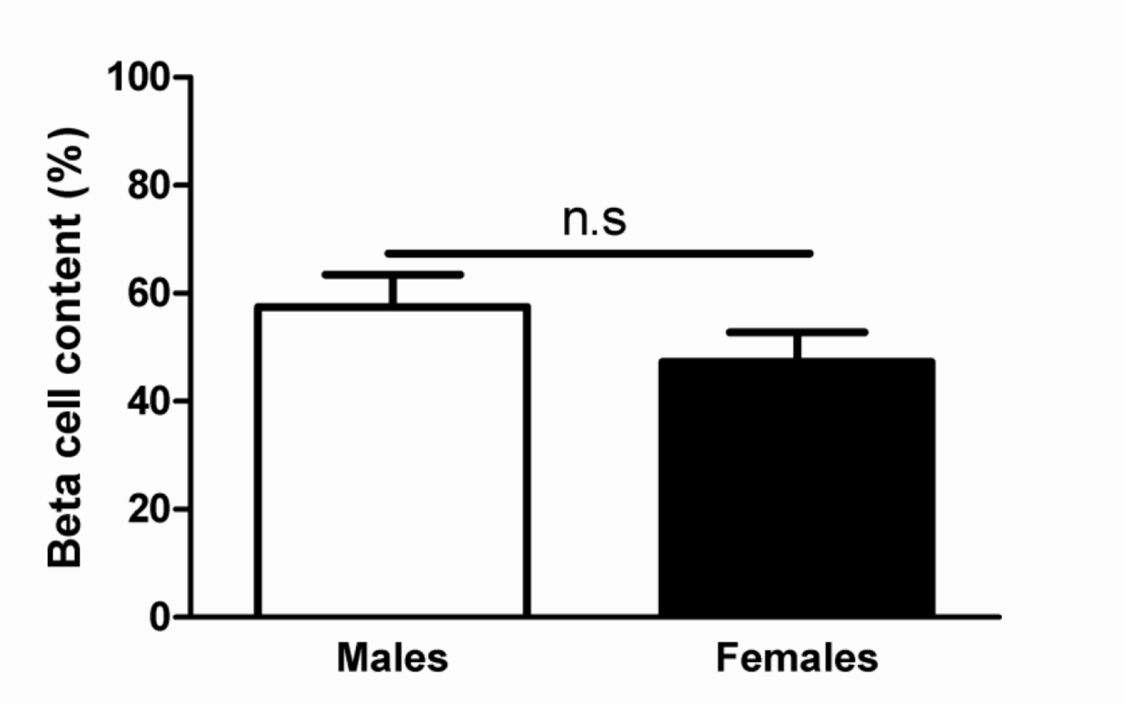

Supplement: Additional file 1: — Sex does not affect the relative β-cell number in human pancreatic islets. Analysis of β-cell number with transmission electron microscopy showed no difference between male and female human pancreatic islets. P =0.29 based on a Mann-Whitney U test. Data are presented as mean ± standard error of the mean. [file 13059_2014_522_MOESM1_ESM.pdf]
