# Supplementary material for: Sex differences in the genome-wide DNA methylation pattern and impact on gene expression, microRNA levels and insulin secretion in human pancreatic islets
Source: Genome Biol. 2014 Dec 3;15(12):522. doi: 10.1186/s13059-014-0522-z (PMC4256841; doi:10.1186/s13059-014-0522-z)
Supplement: Additional file 10: — KEGG pathway analysis result. Results from KEGG pathway analysis of genes with differential methylation in males and females for the autosomal chromosome genes. [file 13059_2014_522_MOESM10_ESM.pdf]

Additional file 10: Results from KEGG pathway analysis of genes with a differential methylation in males and females for the autosomal chromosome genes.

Higher DNA methylation in Males						
**Pathway (total number of genes in the pathway)**	**Observed number of genes**	**Expected number of genes**	**Ratio Observed/ expected**	**Raw *p*-value**	**Adj. *p*-value**	**Observed genes**
Adipocytokine signaling pathway (68)	3	0.26	11.43	0.0023	0.0299	*IRS1*, *PRKAG2*, *NPY*
Higher DNA methylation in Females						
**Pathway (total number of genes in the pathway)**	**Observed number of genes**	**Expected number of genes**	**Ratio Observed/ expected**	**Raw *p*-value**	**Adj. *p*-value**	**Observed genes**
Cell adhesion molecules (CAMs) (130)	5	0.86	5.83	0.0017	0.0408	*HLA-G*, *ITGAL*, *HLA-C*, *CNTNAP2*, *NRXN1*
