# Supplementary material for: Sex differences in the genome-wide DNA methylation pattern and impact on gene expression, microRNA levels and insulin secretion in human pancreatic islets
Source: Genome Biol. 2014 Dec 3;15(12):522. doi: 10.1186/s13059-014-0522-z (PMC4256841; doi:10.1186/s13059-014-0522-z)
Supplement: Additional file 22: — Putative transcription factor binding sites in the SPESP1 promoter. List of transcription factors with known repressive effect and putative binding sites in the SPESP1 promoter. [file 13059_2014_522_MOESM22_ESM.pdf]

Additional file 22: Putative transcription factor binding sites in the SPESP1 promoter. List of transcription factors with known repressive effect and putative binding sites in the SPESP1 promoter.

Transcription factor	Sequence (forward strand sequence shown)*	Strand	HhaI site in or close to binding site**	SssI site in or close to binding site***
AML1a[1]	tttTGAGGTgga	Reverse	No	No
	ctgACCTCAggt	Reverse	No	No
	acaACCTCAtgc	Reverse	No	No
	tctTGGGGTgct	Forward	No	No
	cgcTGTGGTaat	Forward	No	Yes
CDXA[2]	gaaTACAAATcta	Reverse	No	No
	gagTTTTCTGGaat	Forward	No	No
	tttAAAAATAacc	Forward	No	No
	ataCAGAAAAAcag	Reverse	No	No
	tttTATTTTTatt	Reverse	No	No
	tttATTTATTttt	Forward	No	No
	attTATTTTTttg	Reverse	No	No
	ttgTATTTTTagt	Reverse	No	No
	atcTATTAGTccc	Reverse	No	No
	gttTTTAATGccc	Reverse	No	No
CREB[3]	tccTGACCTCAggt	Forward	No	No
	gatTACAGGAGTGAGCCacc	Forward	No	No
DELTAEF1[4]	tggCCCAGGTGCTAagc	Reverse	No	No
	tttTTGAGGTGGAGtct	Reverse	No	No
	gacCTCAGGTGGTCcgc	Reverse	No	Yes
E2F[5]	ctgGCGAGAAAtcg	Reverse	No	Yes
	cccTTTCACGCtgt	Forward	No	Yes
ETS1[6]	ccaCCCGGAACTCggg	Forward	No	Yes
	tgcTACAGGAAGAGTgag	Forward	No	No
	cctTGCTTCCTGAggc	Reverse	No	No
	cccTTGCTTCCTGAGGCaga	Reverse	No	No
	ctcGCCGGAACTAtgc	Forward	No	Yes
	accCGCTTCCGGGggt	Reverse	No	Yes
GATA1[7]	tggCACGATCTCGgct	Reverse	No	Yes
	cctACCCCTATCTTTTTaag	Reverse	No	No
	tgcCAGGTTATCTGCCTctc	Reverse	No	No
	*gcgC*AGGATGGGCcga	Forward	Yes	Yes
	*gcgCGC*GATCCCCcgc	Forward	Yes	Yes
HFH2[8]	tgcGAATTTTTATTTtta	Forward	No	Yes
	tatTTTTATTTATTTTttt	Forward	No	No
	tttATTTATTTTTTTgag	Forward	No	No
LYF1[9]	tccTTTGGGATGtgc	Forward	No	No
	cagTCTCCCAAGtag	Reverse	No	No

	cggCCTCCCAAAgtg	Reverse	No	Yes
	tgcCCTCCCAAGccc	Reverse	No	No
MZF1[10]	cccTGCGGGGAggc	Forward	No	Yes
	gccAGCGGGGAcgg	Forward	No	Yes
	agcTCCCCCCAact	Reverse	No	No
	tagTCCCCAAAaca	Reverse	No	No
	agcTCCCCACCcgc	Reverse	No	Yes
	gctTCCCCCACtac	Reverse	No	No
	cttCCCCCACTaca	Reverse	No	No
	cgaTCCCCCGCccc	Reverse	No	Yes
	ctcTCCCCTCCccc	Reverse	No	No
	cccTCCCCCATgcc	Reverse	No	No
	cgcTCTCCCCTCCCCCatg	Reverse	No	Yes
P300[11]	ggtCTCGAACTCCTGACctc	Reverse	No	Yes
PBX1[12]	attTTTATTTATttt	Reverse	No	No
REL[13]	gggGTGGGTTTCCact	Forward	No	No
	cctGCACGCGGtcc	Reverse	No	Yes
RREB1[14]	agtCCCCAAAACAACCTcat	Forward	No	No
SP1[15]	tccGGGGGTGGGTttc	Forward	No	Yes
	gaaGGGGCGGGGCggg	Forward	No	Yes
	ggcGGGGCGGG*GCgc*g	Forward	Yes	Yes
	gatCCCCCGCCCCCtg	Reverse	No	Yes
	ggaGGGGCGGGCCttg	Forward	No	Yes
SRY[16]	gagTGAGTTTtct	Reverse	No	No
	acaAATCTAAtca	Forward	No	No
	gaaAAACAGAttc	Forward	No	No
	ccaAAACAACctc	Forward	No	No
	gtcAAAGAAAtgg	Forward	No	No
	gaaAAACAAAaac	Forward	No	No
	caaAAACACCcaa	Forward	No	No
	ttgAAAAACAAAAACacc	Forward	No	No

* Capital letters indicate the putative transcription factor binding site, while lower case letters indicate the 3 bases immediately outside the binding site

** Indicated by *italic* letters in sequences

*** Indicated by underscored letters in sequences

References

1. Vu LP, Perna F, Wang L, Voza F, Figueroa ME, Tempst P, Erdjument-Bromage H, Gao R, Chen S, Paietta E, et al: **PRMT4 blocks myeloid differentiation by assembling a methyl-RUNX1-dependent repressor complex.** *Cell Rep* 2013, **5:**1625-1638.
2. Rath B, Pandey RS, Debata PR, Maruyama N, Supakar PC: **Molecular characterization of senescence marker protein-30 gene promoter: identification of repressor elements and functional nuclear factor binding sites.** *BMC Mol Biol* 2008, **9:**43.
3. Sakai T, Kidokoro Y: **Overexpression of a CREB repressor isoform enhances the female sexual receptivity in Drosophila.** *Behav Genet* 2002, **32:**413-422.

4. Eger A, Aigner K, Sonderegger S, Dampier B, Oehler S, Schreiber M, Berx G, Cano A, Beug H, Foisner R: **DeltaEF1 is a transcriptional repressor of E-cadherin and regulates epithelial plasticity in breast cancer cells.** *Oncogene* 2005, **24:**2375-2385.
5. Brehm A, Miska EA, McCance DJ, Reid JL, Bannister AJ, Kouzarides T: **Retinoblastoma protein recruits histone deacetylase to repress transcription.** *Nature* 1998, **391:**597-601.
6. Li R, Pei H, Watson DK, Papas TS: **EAP1/Daxx interacts with ETS1 and represses transcriptional activation of ETS1 target genes.** *Oncogene* 2000, **19:**745-753.
7. Kang HJ, Voleti B, Hajszan T, Rajkowska G, Stockmeier CA, Licznerski P, Lepack A, Majik MS, Jeong LS, Banasr M, et al: **Decreased expression of synapse-related genes and loss of synapses in major depressive disorder.** *Nat Med* 2012, **18:**1413-1417.
8. Sutton J, Costa R, Klug M, Field L, Xu D, Largaespada DA, Fletcher CF, Jenkins NA, Copeland NG, Klemsz M, Hromas R: **Genesis, a winged helix transcriptional repressor with expression restricted to embryonic stem cells.** *J Biol Chem* 1996, **271:**23126-23133.
9. Trinh LA, Ferrini R, Cobb BS, Weinmann AS, Hahm K, Ernst P, Garraway IP, Merkenschlager M, Smale ST: **Down-regulation of TDT transcription in CD4(+)CD8(+) thymocytes by Ikaros proteins in direct competition with an Ets activator.** *Genes Dev* 2001, **15:**1817-1832.
10. Yan QW, Reed E, Zhong XS, Thornton K, Guo Y, Yu JJ: **MZF1 possesses a repressively regulatory function in ERCC1 expression.** *Biochem Pharmacol* 2006, **71:**761-771.
11. Girdwood D, Bumpass D, Vaughan OA, Thain A, Anderson LA, Snowden AW, Garcia-Wilson E, Perkins ND, Hay RT: **P300 transcriptional repression is mediated by SUMO modification.** *Mol Cell* 2003, **11:**1043-1054.
12. Koss M, Bolze A, Brendolan A, Saggese M, Capellini TD, Bojilova E, Boisson B, Prall OW, Elliott DA, Solloway M, et al: **Congenital asplenia in mice and humans with mutations in a Pbx/Nkx2-5/p15 module.** *Dev Cell* 2012, **22:**913-926.
13. Fu T, Li P, Wang H, He Y, Luo D, Zhang A, Tong W, Zhang L, Liu B, Hu C: **c-Rel is a transcriptional repressor of EPHB2 in colorectal cancer.** *J Pathol* 2009, **219:**103-113.
14. Flajollet S, Poras I, Carosella ED, Moreau P: **RREB-1 is a transcriptional repressor of HLA-G.** *J Immunol* 2009, **183:**6948-6959.
15. Zaid A, Hodny Z, Li R, Nelson BD: **Sp1 acts as a repressor of the human adenine nucleotide translocase-2 (ANT2) promoter.** *Eur J Biochem* 2001, **268:**5497-5503.
16. Peng H, Ivanov AV, Oh HJ, Lau YF, Rauscher FJ, 3rd: **Epigenetic gene silencing by the SRY protein is mediated by a KRAB-O protein that recruits the KAP1 co-repressor machinery.** *J Biol Chem* 2009, **284:**35670-35680.
