# Supplementary material for: Sex differences in the genome-wide DNA methylation pattern and impact on gene expression, microRNA levels and insulin secretion in human pancreatic islets
Source: Genome Biol. 2014 Dec 3;15(12):522. doi: 10.1186/s13059-014-0522-z (PMC4256841; doi:10.1186/s13059-014-0522-z)
Supplement: Additional file 23: — Assay information for technical validation of the Infinium HumanMethylation450 BeadChip (Illumina) DNA methylation data. Table showing the assays used for technical validation of the Infinium HumanMethylation450 BeadChip (Illumina) using pre-designed PyroSequencing (Qiagen) assays. Position is based on genome build 37. [file 13059_2014_522_MOESM23_ESM.pdf]

Additional file 23: Assay information for technical validation of the Infinium HumanMethylation450 BeadChip (Illumina) DNA methylation data. Table showing the assays used for technical validation of the Infinium HumanMethylation450 BeadChip (Illumina) using pre-designed PyroSequencing (Qiagen) assays. Position is based on genome build 37.

**Infinium HumanMethylation450 BeadChip data**				**PysoSequencing assays**
**Illumina probe ID**	**Chr**	**Position**	**Gene**	**Pre-designed assay ID**
cg27483305	X	119077757	*NKAP*	Hs_CG27483305_01_PM_Q96
cg05688478	X	128789194	*APLN*	Hs_CG05688478_01_PM_Q96
cg13808071	2	11679872	*GREB1*	Hs_CG13808071_02_PM_Q96
