# Supplementary material for: Sex differences in the genome-wide DNA methylation pattern and impact on gene expression, microRNA levels and insulin secretion in human pancreatic islets
Source: Genome Biol. 2014 Dec 3;15(12):522. doi: 10.1186/s13059-014-0522-z (PMC4256841; doi:10.1186/s13059-014-0522-z)
Supplement: Additional file 24: — Sequences for the NKAP and SPESP1 promoters used for luciferase assay. We cloned 1,500 bp fragments of the NKAP and SPESP1promoters covering CpG sites that exhibited differential DNA methylation in female versus male islets into a CpG-free luciferase reporter vector for the luciferase assay. [file 13059_2014_522_MOESM24_ESM.pdf]

Additional file 24: Sequences for the *NKAP* and *SPESP1* promoters used for luciferase assay. 1500bp fragments of the respective promoters covering several CpG sites that exhibited differential DNA methylation in female versus male islets were cloned into a CpG-free luciferase reporter vector for the luciferase assay.

NKAP

5′-

GAGTGAGAGTACACTATTTTTTTTTGGAAACACAGTCTCACTCTGTTGCC
CAGGCTGGAGTGCAGTGGTGCAATCTCACTCTCGGCTCACTGCAACCTCA
GCCTCCCGGGTTCAAGCGATTCTCGTGCCTCAGCCACCCTAGTAGCTGAG
ATTACAGGCACGTGCCACCAGGCCCAGCTAATTTTTGTATTTTTAGTGGA
GACAGGGTTTCGCCATGTTGGCCAGGCTGGTCTCAAACTCCTGACCTCAA
GTGATCCACCCGCCTCTGCCTCCCAAAGTGCTGGGATTACAGACGTGAGC
CACCATACCAGGCCGAGAGTACACTTAACAAAAATGTATTGTGCTCAGAT
AATGGACACCTTAAATACCCTGACGTCATCACTACACATTATATACATGT
AACAAAATTTCACATGTATCCCATAGATTTGTACAAATTTAAAAAATAAT
AAAAAATTAGCTGTGCATGATGGCACATGTCTGTAATCCCAGCTACTCAG
GAGGCTGAGGCAGGAGAATCACTTGAACCTGGGAGGCGGAGGTTGCAGTG
AGCCAGGATAGCCACTGCACTCCAGCCTGGATGACAGAGGGAGCCCTTGT
CTCAAAAATAAAAAAATAAAAAATAAAATGACACTTTTTGGGGAAAAAAA
TACAAGCCAGACGCAGTGGCTCACACCTATAATCCTAGCACTTTGGGAGG
CCGAGGCTGGCGGATCGCTTGAGCCCAGGAGTTCAAGACCACCTGGGCAA
CAAGGTGAAACCCTGTTTCCCCCGACCCCCCTCAAAAAAAAAACTACCCA
GGCTTGGTGTCGCATGCGCCTGTAATCCCAGCTATTCAGGAGGCTGAGGT
GGGAGGATAGCTTGAGCCCGGGGAGGCGGAGGTTTGCAGGGAGCTGAGAT
CTTGCCACTGTACTCCATCCTGGGCGACAGAGTGAGCAGAGTGAGTTCCA
GTCTCAAAAAAAAAAAACCTCCTTTTGCTGGGGCATATTGGTGGAGACGG
TGGCTAGGGGATAAGTATGCAGTTTTTCTCTAGGCGGGAAACTTAATTGA
AGTTTATCAGGCGCGAGTCATTAACAAAATTTGCGCCCTCTAAGCTAACA
CGGCAATATGGGGCTTAGACACCATAGCTAATCCCCAGTCGCGAAAAACC
CTCCCGATTTGCTTGGGATTGGAAAGAAAAAGAAAATACACATTCATACT
TTCATACATGACATACACACACACACACACATTTATATATAAATACACAC
AGAAAGCACAGGAAACACAAGCAGAATTAACTGTCTTGTTCATACGGCCA
CTCAGTGAAATGTATACTGTATATTGTGTTTGAAATATATACTTCCCTTC
AAGTGTTTCAAGTTCAAGGCTGAGAAAGAACTTGGTCTCTTGCTTAGACT
TGAGGAGCCTCACTCGGAACTGGAAGGATGCACGCAATCTAGTTGCCCCA
CCGGCTTATTTATATTGATGCACACATCGCTTCCTGTCGGTGACGACCGC

-3′

SPESP1

5′-

CAGGGAGCGGCGCTCCTCAGGGAGGCTCAGGCATGGCAGGCTGCAGGTCC
CGAGCCCTGCCCTGCGGGGAGGCAGCTAAGGCCTGGCGAGAAATCGAGCA
CAGCAGCTCCTGGCCCAGGTGCTAAGCTCCCCCCAACTGCCCGTGGCCAG
CGGGGACGGCCGGCCGCGCTGAGTGCCGGGCCCACCGAGCCCGCCGAGCC
CACGCCCACCCGGAACTCGGGCTGGCCCGCAAGCGCCGTGCGCAGTCTCA
GTTTCCGCCGGCGCCTCTAGTCTTGCTTCCCTCCAGTCCATTCTTCATGC
TACAGGAAGAGTGAGTTTTCTGGAATACAAATCTAATCACAACCGTTCCC
AGCTCAAAACCTAGCAGTACTCTAGTACTGGTGCCTCAGACTAAAATCCA
AGCCCCTTGCTTCCTGAGGCAGAGATGAGTGTACGTGAATTTTTAAAAAT
AACCATACAGAAAAACAGATTCTTTGGGATGTGCAGTTCTGCGAATTTTT
ATTTTTATTTATTTTTTTGAGGTGGAGTCTTGCTCTGTCGCCCAGGCTGG

AGTGCAGTGGCACGATCTCGGCTCACTGCAACCTCTGCTTCCCGGGTTCA
AGTGATTCTCCTGCCTCAGTCTCCCAAGTAGCTGGGACTACAGGCGTGTG
CCACCACCCACGCCTGGCTAATTTTGTATTTTTAGTAGCCACGAAGTTTC
ACTATGTTGGTCAGGCTGGTCTCGAACTCCTGACCTCAGGTGGTCCGCCC
ACCTCGGCCTCCCAAAGTGCTGGGATTACAGGAGTGAGCCACCGTGCCCG
GCCTTCTGTGAATTTTAACACATGTATAGATTCTTGTGGCCACTGCCATC
TATTAGTCCCCAAAACAACCTCATGCTATCCTTTCATAGTCGTACTCTCC
CTCATCCCTACCCCTATCTTTTTAAGAAGGTCAAAGAAATGGAATGTGAT
CTTTTGAAAAACAAAAACACCCAAGCCCCTTAGGGCGTTTTTAATGCCCT
TGCCTACCTCTCCTCCCTGTCCTCCAACAACTGCCCTCCCAAGCCCCACA
ATGCAACTCGCCGGAACTATGCTCAGTTCTTGGGGTGCTCCAAGCTGTCC
TCCACTTAGCCAAGCTCCCCACCCGCTTCCGGGGGTGGGTTTCCACTGTG
CACAACAGTACCTAGCGCTTCCCCCACTACAGCCCCCTTTCACGCTGTGG
TAATTGCCAGGTTATCTGCCTCTCCCATTACAGTTCCTTCCTCACAGGGA
CCGCGTTTAAACCACGGCGGTATCCCTACCGCGCAGGATGGGCCGAGCCC
CGCACGCCCCGAGGCAGCACCCCGCACGCAGTAGCCGGGCGTCGAGGCGT
TCCCAGCCCACACCAGGCGCGGACCTACGAAGGCGGAGCGAGCATGCGCA
CTAGGAAGGGGCGGGGCGGGGCGCGCGCGATCCCCCGCCCCCTGCACGCG
GTCCCAGGAGGGGCGGGCCTTGGCCGCTCGCTCTCCCCTCCCCCATGCCG
-3′
